# The First Physician in Massachusetts

**Published:** 1869-04

**Authors:** 


The First Physician in Massachusetts.—Dr. Samuel Fuller, the physician of the Mayflower, was the first disciple of Galen mentioned in the history of Massachusetts.

